# MMF/MPA Is the Main Mediator of a Delayed Humoral Response With Reduced Antibody Decline in Kidney Transplant Recipients After SARS-CoV-2 mRNA Vaccination

**DOI:** 10.3389/fmed.2022.928542

**Published:** 2022-07-07

**Authors:** Julian Stumpf, Torsten Siepmann, Jörg Schwöbel, Grit Glombig, Alexander Paliege, Anne Steglich, Florian Gembardt, Friederike Kessel, Hannah Kröger, Patrick Arndt, Jan Sradnick, Kerstin Frank, Anna Klimova, René Mauer, Torsten Tonn, Christian Hugo

**Affiliations:** ^1^Medizinische Klinik und Poliklinik III, Universitätsklinikum, Carl Gustav Carus, Technische Universität Dresden, Dresden, Germany; ^2^KfH-Nierenzentrum Dresden, Dresden, Germany; ^3^KfH-Nierenzentrum am Klinikum Chemnitz, Krankenhaus Küchwald, Chemnitz, Germany; ^4^Dialysezentrum Chemnitz, Chemnitz, Germany; ^5^KfH-Nierenzentrum am Klinikum St. Georg, Leipzig, Germany; ^6^Institut für Transfusionsmedizin Plauen, DRK-Blutspendedienst Nord-Ost gemeinnützige GmbH, Plauen, Germany; ^7^National Center for Tumor Diseases (NCT) Dresden, Dresden, Germany; ^8^Faculty of Medicine Carl Gustav Carus, Institute for Medical Informatics and Biometry (IMB), Technische Universität Dresden, Dresden, Germany; ^9^Institute for Transfusion Medicine, German Red Cross Blood Donation Service North-East, Dresden, Germany; ^10^Faculty of Medicine Carl Gustav Carus, Transfusion Medicine, Technische Universität Dresden, Dresden, Germany

**Keywords:** vaccination, kidney transplant recipients, SARS-CoV-2, humoral response, mycophenolic acid, clinical decision making, guidelines

## Abstract

Kidney transplant recipients (KTR) show significantly lower seroconversion rates after SARS-CoV-2 mRNA vaccination compared to dialysis patients (DP). Mycophenolate mofetil or mycophenolic acid (MMF/MPA) in particular has been identified as a risk factor for seroconversion failure. While the majority of all KTR worldwide receive MMF/MPA for immunosuppressive therapy, its impact on antibody decline in seroconverted KTR still remains unclear. In an observational study (NCT04799808), we investigated whether 132 seroconverted KTR (anti-spike S1 IgG or IgA positive after 2 vaccinations) show a more rapid antibody decline with MMF/MPA than those without this medication. A total of 2 months after mRNA vaccination, average anti-spike S1 IgG levels of KTR with MMF/MPA were lower than without (*p* = 0.001), while no differences between these two groups were observed after 6 months (*p* = 0.366). Similar results were obtained for anti-RBD IgG antibodies (T2 *p* = 0.003 and T3 *p* = 0.135). The probability of severe IgG decline with MMF/MPA was three times lower than without (*p* = 0.003, OR 0.236, 95% CI 0.091–0.609). In the multivariate analysis, neither immunosuppressants, such as calcineurin inhibitors, mTOR inhibitors (mTOR-I; mechanistic target of rapamycin), glucocorticoids, nor vaccine type, sex, or age showed a significant influence on IgG titer decline between 2 and 6 months. For the decision on additional booster vaccinations, we consider immunosurveillance to be needed as an integral part of renal transplant follow-up after SARS-CoV-2 mRNA vaccination. Not only the lack of seroconversion but also the peak and titer decline of the specific IgG and RBD IgG antibody formation after two mRNA vaccinations is significantly influenced by MMF/MPA.

## Introduction

Immunosuppressive therapy in kidney transplant recipients (KTR) is the main determinant for highly impaired seroconversion rates compared to the normal population after SARS-CoV-2 mRNA vaccination (1–3). Hereby, studies including our Dia-Vacc study identified the anti-metabolite MMF/MPA (besides belatacept) as the critical immunosuppressive drug type being associated with seroconversion failure at 2 months after SARS-CoV-2 vaccination in KTR (1–3). During 6 months of follow-up investigations, seroconverted KTR [compared to medical personnel (MP)] were at risk for a strong decline in IgG and RBD-IgG antibodies but neither IgA antibodies nor cellular immunity (4). Hereby, antibody titers of KTR peaked at a lower level, and pronounced antibody decline was mixed with an increasing IgG or RBD-IgG response in at least 15% of patients. Despite MMF/MPA being given to the majority of all organ transplant recipients worldwide, its influence on antibody decline in seroconverted transplant recipients after SARS-CoV-2 mRNA vaccination is unclear. According to the pre-existing data on the impact of MMF/MPA on vaccination-related seroconversion and antibody formation, we hypothesized that MMF/MPA treatment may also lead to a pronounced antibody decline within additional 4 months of follow-up after seroconversion at the 8-week time point after mRNA vaccination starts in 132 KTR of the DIA-Vacc cohort.

## Methods

### Study Design

**Figure 1:**
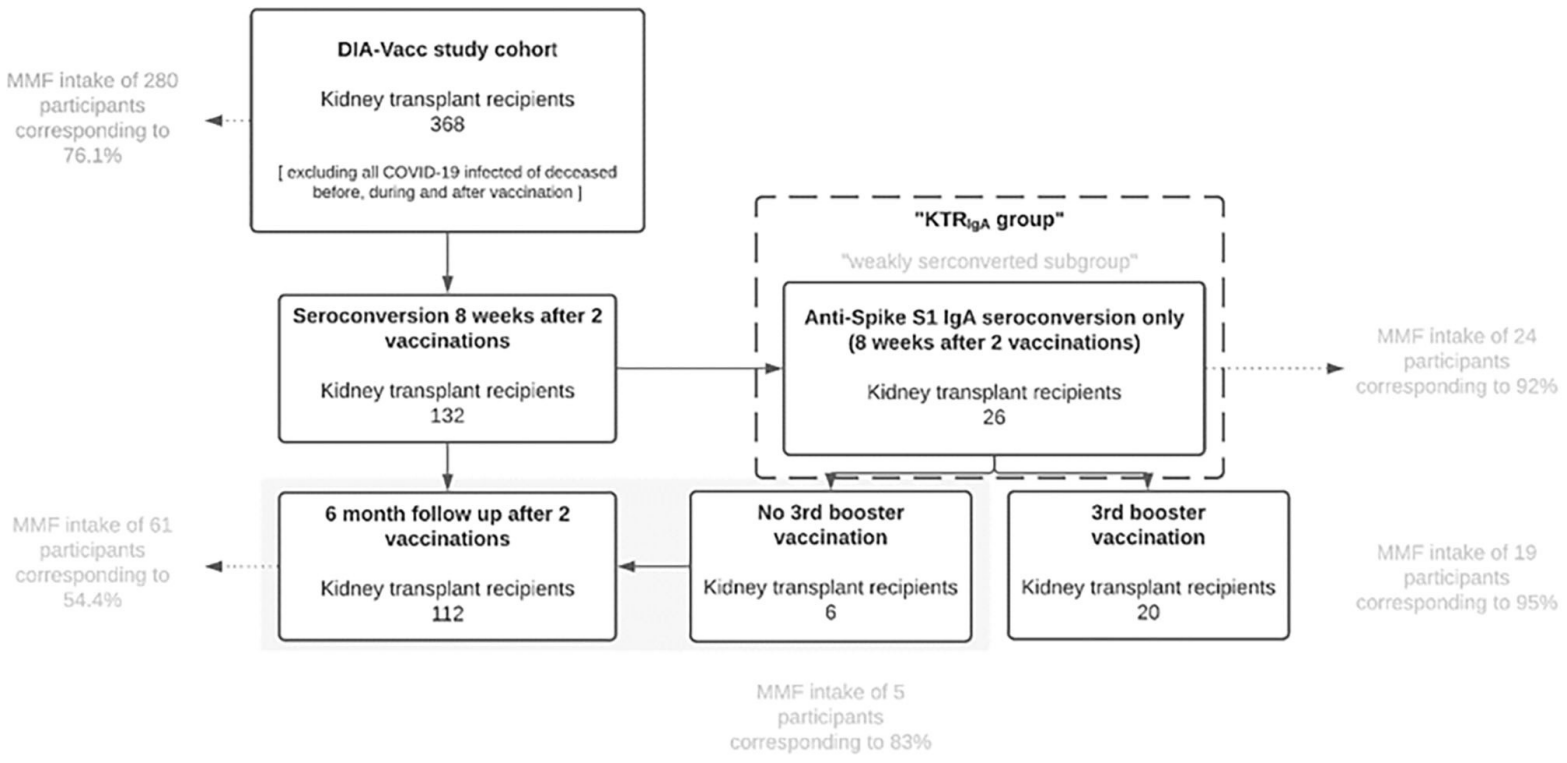
KTR_IgA_ group. KTR, Kidney Transplant Recipients; MMF, mycophenolate mofetil or mycophenolic acid.

In the 2- (T2) and 6-month (T3) evaluation (Supplementary Figure 1) of the prospective DIA-Vacc (1) observational study (NCT04799808), we analyzed the specific cellular (interferon-γ release assay) and humoral immune responses after 2x SARS-CoV-2 mRNA without a third vaccination in 112 out of 132 KTR (see Supplementary Table 1) with seroconversion (de novo IgA or IgG antibody positivity by ELISA (enzyme-linked immunosorbent assay), KTR_112_). In addition, 26 out of 132 KTR with seroconversion represent a separate group of IgA de novo positive only, but IgG negative KTR_IgA_ at T2 (Figure 1, also referred to as “weakly seroconverted”). In 20 out of 26 cases of this KTR_IgA_ group by unanticipated, individual decision of the dialysis centers (procedure legally permitted in Germany), an additional third vaccination was done at 4.2 ± 1 months. At T0 (start of vaccination), T2, and T3, SARS-CoV-2-specific IgG or IgA antibody responses to Spike S1 protein and antibodies to the receptor binding domain (RBD-IgG) at T2 orT3 were assessed in all study participants (1). Titer levels and changes were classified differently depending on what was being looked at. For the assessment and comparison of subcohorts defined by a response of comparable expression (i.e., titer decrease to comparable absolute values), levels and corresponding limits were defined (see 2.4 Interval classification in levels). For the overall assessment and comparisons with regard to relative changes defined as increasing, equal, or decreasing titers, ranges were formed. For the latter, a range of 20% for the T3 compared to T2 change of antibody and interferon-γ release assay (IGRA) titers/values (increased or equal or decreased) was used and the percentage of patients within each range was calculated (Table 1). In addition, the antibody time course was analyzed on the interval scale. The detectable ranges of anti-spike S1-IgG and RBD-IgG antibody values were categorized into five intervals, labeled from 0 to 4 (referred to as “levels” in the data analysis), and the change in levels, varying from −4 to +4, was calculated for each patient. Level decreases from T2 to T3 by at least two units were defined as a strong decline (1).

**Table 1:** Immune response rates 6 months after vaccination (T3) compared to T2 in the seroconverted Kidney transplant recipients (KTR)_112_ cohort.

**Variable**	**Category**	**KTR without MMF/MPA**	**KTR with MMF/MPA**	***p*-value**
**Patient number**	***n***	51	61	
**Humoral responses**				
IgG-Ab or IgA-Ab Spike S1 positive	*n* of total *n* (%)	45 / 51 (88.2 %)	53 / 61 (86.9 %)	1
IgA-Ab Spike S1 positive	*n* of total *n* (%)	29 / 51 (56.8 %)	37 / 61 (60.7 %)	0.831
IgA-Ab Spike S1 increasing	*n* of total *n* (%)	2 / 51 (3.9 %)	2 / 61 (3.3 %)	1
IgA-Ab Spike S1 equal	*n* of total *n* (%)	0 / 51 (0 %)	2 / 61 (3.3 %)	0.556
IgA-Ab Spike S1 decreasing	*n* of total *n* (%)	49 / 51 (96.1 %)	57 / 61 (93.4 %)	0.845
IgG-Ab Spike S1 positive	*n* of total *n* (%)	42 / 51 (82.4 %)	52 / 61 (85.3 %)	0.875
IgG-Ab Spike S1 increasing	*n* of total *n* (%)	2 / 51 (3.9 %)	15 / 61 (24.6 %)	0.006
IgG-Ab Spike S1 equal	*n* of total *n* (%)	14 / 51 (27.5 %)	17 / 61 (27.9 %)	1
IgG-Ab Spike S1 decreasing	*n* of total *n* (%)	35 / 51 (68.6 %)	29 / 61 (47.6 %)	0.04
RBD-IgG positive	*n* of total *n* (%)	35 / 51 (68.6 %)	31 / 61 (50.8 %)	0.086
RBD-IgG increasing	*n* of total *n* (%)	1 / 50 (2.0 %)	11 / 56 (19.6 %)	0.011
RBD-IgG equal	*n* of total *n* (%)	18 / 50 (36.0 %)	18 / 56 (32.1 %)	0.831
RBD-IgG decreasing	*n* of total *n* (%)	31 / 50 (62.0 %)	27 / 56 (48.2 %)	0.219
RBD-IgG de novo	*n* of total *n* (%)	1 / 50 (2.0 %)	5 / 56 (8.9 %)	0.263
**Interferon-γ** **release assay (IGRA)– T-cellular response**				
IGRA positive	*n* of total *n* (%)	8 / 20 (40.0 %)	7 / 22 (31.8 %)	0.818
IGRA increasing	*n* of total *n* (%)	6 / 18 (33.3 %)	6 / 17 (35.3 %)	1
IGRA equal	*n* of total *n* (%)	1 / 18 (5.6 %)	0 / 17 (0 %)	1
IGRA decreasing	*n* of total *n* (%)	11 / 18 (61.1 %)	11 / 17 (64.7 %)	1

*MMF/MPA, mycophenolate mofetil or mycophenolic acid*.

*In Table 1 using 20% as a margin, the time course of antibody or IGRA titers at T3 compared to T2 time point were categorized into increased (>20%), equal (within 20% range), and decreased (<20%). De novo positivity on T3 means that despite overall seroconversion on T2 (for either IgA or IgG antibodies), the value for RBD-IgG was negative on T2 but positive on T3. Humoral vaccination responses were assessed as positive when de novo production of the antibody to the Spike S1 (IgA or IgG) protein or RBD (IgG) subunit was above the positivity level. A positive T-cellular response to vaccination as assessed by interferon-γ release assay (IGRA) turned from a negative result on T0 to positive on T3, respectively (≥100 mIU/ml, as being recommended by the manufacturers)*.

For this evaluation, all participants with asymptomatic* or documented symptomatic** COVID-19 disease before and during vaccination up to T3 (6 months) were excluded.

^*^Asymptomatic COVID-19 disease definition—neither knowledge nor symptoms of COVID-19 disease, but IgG-antibody reaction to nucleocapsid (T0, T2, or T3) or to the Spike protein subunit S1 (only T0) of the SARS-CoV-2 virus is positive.

^**^Symptomatic COVID-19 disease definition—SARS-CoV-2 PCR positive patients with clinical symptoms.

### Background Study Design

In the original investigator-driven, non-interventional, prospective, observational DIA-Vacc study (1), the first 26 out of all 36 regional nephrology centers were recruited. Further centers could not be considered due to funding restrictions. A total of 3,101 participants were enrolled to explore the time course of a specific cellular response or/and humoral seroconversion to disease and/or SARS-CoV-2 vaccination in MP, DP, and 368 KTR (see Figure 1). To report clean humoral seroconversion rates, as reported here, a “pure vaccination cohort” was created excluding retrospectively all symptomatically and asymptomatically COVID-19 infected or deceased participants before, during, and after vaccination (up to T2). For further description, see elsewhere (1). Another cohort called the “clinical vaccination cohort” consists of the “pure vaccination” cohort but includes all participants who experienced symptomatic or asymptomatic COVID-19 disease (or death) strictly during or after vaccination to assess the clinical outcome of vaccination. The study start (T0) was immediately before the first vaccination. Further monitoring of time points is described elsewhere (1). By vaccine availability, initially, only the first four dialysis centers were assigned to the vaccination campaign and received BNT162b2 mRNA, while all other following dialysis centers received the mRNA-1273 vaccine for both vaccinations. Neither any dialysis center nor any patient or MP or the study center (Dresden) had a choice or influence regarding the type of vaccine. All dialysis centers were informed *via* simultaneous email from the central vaccination institute, about the start of the vaccination campaign. In all study participants (eligibility if >18 years old and signed informed consent) at T0, T2, and T3, the above-mentioned antibody measurements were done, using Euroimmun ELISAs on Euroimmun analyzers (5–9). To explore the cellular SARS-CoV-2 immune response in subgroups, a SARS-CoV-2 specific interferon-γ release assay (Euroimmun-SARS-CoV-2-IGRA for research use only ET 2606-3003 & EQ 6841-96011,2) was applied (10). The sub-group for the IGRA was formed as follows: the analysis of T cells requires vivid cells. To reach high viability in IGRA samples, the procession should start at <24 h (established at <6 h) after collection. To ensure this high sample quality, four centers in the vicinity of the study coordination center were asked to participate in this sub-group analysis. The selection took into account that the centers treated a sufficient number of transplanted patients and that both vaccines were represented. The exact procedure and analysis are further described elsewhere (1).

### Statistical Analysis

In the descriptive analysis of the main study endpoints, categorical variables were summarized as absolute frequencies or percentages, and continuous variables were summarized using the mean and SD or median and interquartile range (IQR). Time trends in IgG and RBD-IgG responses and between-group differences were analyzed either by the Wilcoxon signed-rank test or the chi-squared test, as appropriate. The analysis of risk factors of patients with a strong antibody decline was carried out using multiple logistic regression. For hypothesis testing, a significance level of 5% (two-sided) was chosen. A Bonferroni correction was applied during *post hoc* testing of group effects. Data analysis was implemented in the R Environment for Statistical Computing (11), version 4.0.4.

### Interval Classification in Levels

In the proposed interval classification, level 0 is assigned to IgG and RBD values below the corresponding positivity threshold [35.2 Binding Antibody Units/ml (BAU/ml) and 35%, respectively], and the remaining values are divided into four intervals of approximately equal length (Supplementary Table 2). These intervals can be used to quantify the change in IgG or RBD-IgG between T2 and T3, where, for example, a positive change corresponds to an increase in IgG or RBD-IgG, respectively, with a change of 4 being the maximum increase. Based on this definition, we referred to any decrease of more than one level (at least two) as a “strong declining response.” The distributions of IgG and RBD-IgG levels at T2 and T3 and their change between T2 and T3 are summarized in Supplementary Figures S2A–F and separately dependent on the use of MMF/MPA.

### Multivariate Analysis

Besides gender, age, and vaccine type, the association between different immunosuppressive drug types of drugs such as calcineurin inhibitors, corticosteroids, mTOR-inhibitors, and MMF/MPA, and strong declining IgG response was explored using a penalized logistic regression model estimated using the elastic net approach (12). Supplementary Figure 3 illustrates a stepwise model selection procedure in which predictors (immunosuppressive 4 drug types) are added to a regression model one at a time, to maximize the goodness-of-fit, assessed from the deviance, given the current number of predictors. The slope of each path in Supplementary Figure 3 changes as a new immunosuppressive drug enters the model. According to this plot, MMF/MPA has the strongest explanatory ability as a single predictor.

### Definition of KTR_IgA_ Group

The KTR_IgA_ group (*n* = 26) is defined as a seroconversion group with de novo IgA positivity without a positive IgG response at T2 after 2x mRNA vaccination (< 35.2 BAU/ml according to manufacturer definition, see also Figure 1). In this group, 24 of 26 (92%) KTR were treated with MMF/MPA since most (20/26) of these had to be excluded due to an unanticipated third vaccination by the dialysis centers despite formal seroconversion. Nineteen of 24 of the MMF/MPA treated KTR_IgA_ group received an additional mRNA vaccine booster between T2 and T3. In contrast, 5 of 24 KTR_IgA_ patients with MMF/MPA were not vaccinated a third time.

## Results

Kidney transplant recipients_112_ group: separation of all 112 KTR in two groups with MMF/MPA (*n* = 61) and without MMF/MPA (*n* = 51) demonstrates that with the exception of immunosuppressive drug types both groups are well matched for patient characteristics (Supplementary Table 1). Multivariate analysis of the KTR_112_ group revealed that MMF/MPA but no other immunosuppressive drug such as calcineurin inhibitors, mTOR-inhibitors, or glucocorticoids significantly influenced vaccination-related IgG anti-spike S1 protein antibody titers and decline between 2 and 6 months (Table 2). While at 2 months, IgG levels of KTR_112_ with MMF/MPA were on average lower than those of KTR_112_ without MMF (*p* = 0.001), at 6 months no differences between these two groups were observed (*p* = 0.366) (Table 3, Figures 2A/B). As it can be observed in Supplementary Figure 3, there is a negative association between taking MMF/MPA and strong IgG decline, that is, patients taking MMF/MPA have a lower chance to experience a strong decline than patients taking other immunosuppressive. An overall decreasing trend occurred in both groups, but KTR_112_ with MMF/MPA were three times less likely to show a strong IgG decline than KTR_112_ without MMF/MPA (*p* = 0.003). A comparable difference for KTR_112_ with and without MMF/MPA was also observed for RBD-IgG: lower values for MMF at T2 (*p* = 0.003) and no significant difference at T3 (*p* = 0.135).

**Table 2:** Multivariate analysis of IgG antibody decline between T2 and T3 in kidney transplant recipients after seroconversion [kidney transplant recipients (KTR)_112_ cohort].

**Risk factor**	**OR**	**95% CI**	***P*-value**
Age	1.034	[0.996, 1.075]	0.083
Sex (Ref. = female)	1.284	[0.504, 3.270]	0.600
Vaccine type (Ref. = mRNA-1273)	1.817	[0.655, 5.041]	0.251
Steroids (Ref. = none)	2.150	[0.845, 5.467]	0.108
CNI (Ref. = none)	1.338	[0.395, 4.533]	0.640
MMF/MPA (Ref. = none)	0.236	[0.091, 0.609]	0.003
mTOR-I (Ref. = none)	0.459	[0.139, 1.517]	0.202

*mRNA-1273 represents Spikevax also called Moderna COVID-19 vaccine; the second vaccine (compared to) is BNT162b2-mRNA which stands for Comirnaty also known as Pfizer-BioNTech COVID-19 vaccine; CNI means calcineurin inhibitors; KTR, Kidney Transplant Recipient; MMF/MPA, mycophenolate mofetil or mycophenolic acid; mTOR-I means mTOR-inhibitors*.

**Table 3:** Antibody and interferon-γ release assay (IGRA) titers 2 (T2) and 6 months (T3) after vaccination in the seroconverted kidney transplant recipients (KTR)_112_ cohort with and without mycophenolate mofetil or mycophenolic acid (MMF/MPA).

**Variable**	**Group**	**Category**	**KTR without MMF/MPA**	**KTR with MMF/MPA**	***p*-value**
**Humoral responses**
IgA-Ab spike S1	T2	Median (interquartile range)	4.3 (2.4–9)	5.2 (1.9–9)	0.827
IgA-Ab spike S1	T3	Median (interquartile range)	1.7 (0.6–3.9)	1.9 (0.8–4.2)	0.568
IgG-Ab spike S1	T2	Median (interquartile range)	384 (215.4–384)	167.8 (84.2–384)	0.001
IgG-Ab spike S1	T3	Median (interquartile range)	149.6 (51.2–375.3)	106.1 (61.1–263.4)	0.366
RBD-IgG-Ab spike S1	T2	Median (interquartile range)	84.8 (55.0–97.9)	59.1 (25.0–88.7)	0.003
RBD-IgG-Ab spike S1	T3	Median (interquartile range)	46.9 (30.7–81.5)	37.9 (17.8–69.3)	0.135
**T-cellular response**
IGRA	T2	Median (interquartile range)	113.3 (13.5–289.6)	79.7 (14.3–454.3)	0.897
IGRA	T3	Median (interquartile range)	75.6 (14.4–176.1)	25.9 (16.1–169.6)	0.876

*KTR, Kidney Transplant Recipient; MMF/MPA, mycophenolate mofetil or mycophenolic acid; Interferon-γ release assay = IGRA*.

**Figure 2A:**
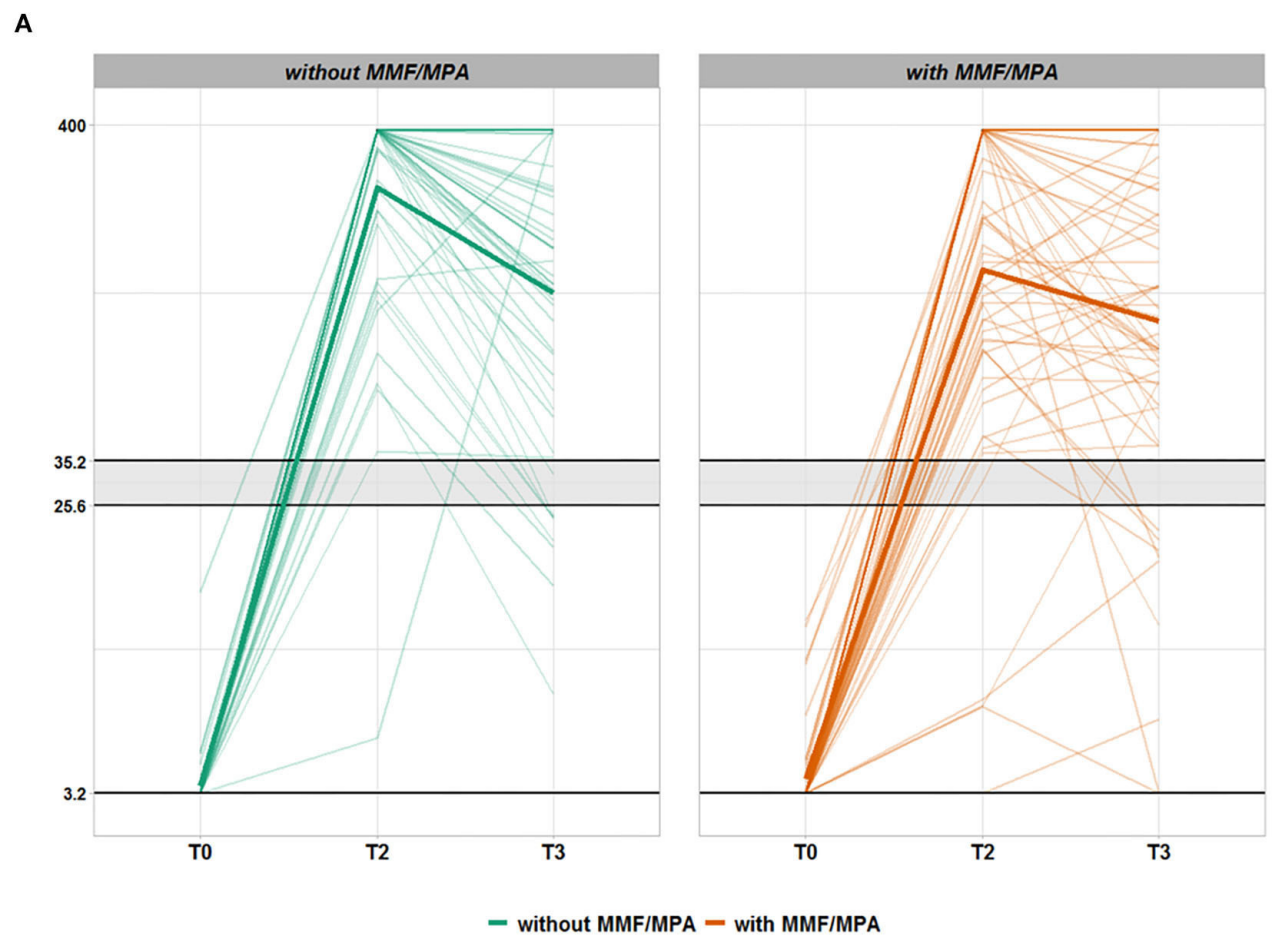
Time course of anti-SARS-CoV-2 IgG antibodies in seroconverted kidney transplant recipients (KTR) without (green) or with (orange) mycophenolate mofetil or mycophenolic acid (MMF/MPA) treatment. Each thin line corresponds to the anti-spike S1 protein IgG antibody values (QuantiVac, Euroimmun) of a study participant from T0 (vaccination start) *via* T2 (8 weeks after vaccination start) to T3 (6 months after vaccination start). KTR being treated without MMF/MPA are represented in green and KTR exposed to MMF/MPA treatment are shown in orange. Only patients with successful de novo seroconversion at T2 (IgA or IgG antibody positivity against the SARS-CoV-2 S1 protein) after 2x mRNA vaccination and without SARS-CoV-2 nucleocapsid (NCP) antibodies were considered. The area shaded gray designates IgG borderline range below positivity level. The vertical axis is depicted on log_10_ scale with the corresponding unit BAU/ml.

**Figure 2B:**
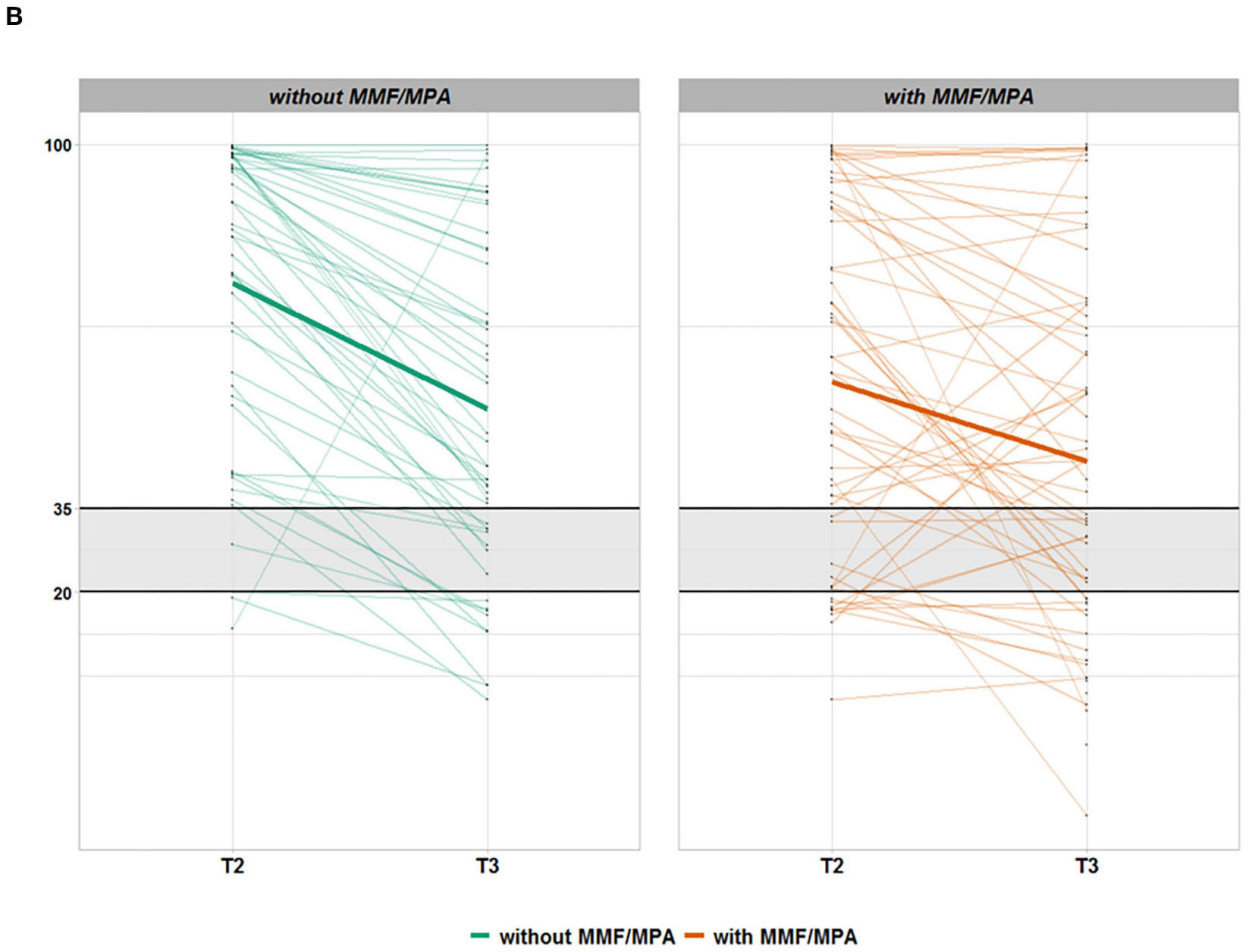
Time course of anti-SARS-CoV-2 RBD-IgG antibodies in seroconverted kidney transplant recipients (KTR) without (green) or with (orange) mycophenolate mofetil or mycophenolic acid (MMF/MPA) treatment. Each thin line corresponds to the anti-spike S1 protein RBD-IgG antibody values (Euroimmun) of a study participant from T2 (8 weeks after vaccination start) to T3 (6 months after vaccination start). KTR being treated without MMF/MPA are represented in green and KTR exposed to MMF/MPA treatment are shown in orange. Only patients with successful de novo seroconversion at T2 (IgA or IgG antibody positivity against the SARS-CoV-2 S1 protein) after 2x mRNA vaccination and without SARS-CoV-2 nucleocapsid (NCP) antibodies were considered. The area shaded gray designates RBD-IgG borderline range below positivity level. The vertical axis is depicted on log_10_ scale with corresponding unit % inhibition.

Using 20% as a margin, only 48% or 48% of patients with MMF/MPA but 69% or 62% of KTR without MMF showed decreased anti-spike S1 IgG or RBD-IgG antibody titers at T3, respectively (Table 1, Figures 2A/B). A total of 25% or 20% of KTR_112_ with MMF/MPA but only 4% or 2% of KTR without MMF/MPA showed IgG or RBD-IgG antibody increases up to T3. This delayed antibody response/increase in patients with MMF/MPA is also reflected by 9% of seroconverted KTR_112_, who are characterized by *de novo* RBD-IgG positivity at T3.

In contrast, anti-spike S1 IgA protein antibody and cellular immunity rates were independent of MMF/MPA use (Table 1).

Only one KTR developed asymptomatic COVID-19 disease with anti-nucleocapsid antibody (NCP) seroconversion.

### Kidney Transplant Recipients_IgA_ Group

Most (20/26) of the “anti-spike S1 IgA antibody seroconverting only” KTR_IgA_ group (IgA but no IgG seroconversion) had to be excluded from the above evaluation due to an unanticipated third vaccination by the dialysis centers despite formal seroconversion. In this subgroup, 24 of 26 (92%) KTR were treated with MMF/MPA further supporting the general MMF/MPA-dependent IgG antibody results of our study. Nineteen of 24 of the MMF/MPA treated KTR_IgA_ patients received an additional mRNA vaccine booster at 4.2 ± 1 month demonstrating a marked IgG (Supplementary Table 3, Supplementary Figure 4) and RBD-IgG (Supplementary Table 3) increases in almost all patients between T2 and T3, respectively. In contrast, 5 of 24 KTR_IgA_ patients with MMF/MPA were not vaccinated a third time and remained at a much lower level of antibody titers T3. Nevertheless, two out of five of these “IgA only seroconverted” patients with MMF/MPA showed a delayed *de novo* positivity of IgG antibodies at T3 without any booster vaccination (Supplementary Figure 4).

## Discussion

The predominantly used immunosuppressive anti-metabolite MMF/MPA impairs both seroconversion rate and IgG and RBD-IgG titers in organ transplant recipients 2 months after SARS-CoV-2 mRNA vaccination (1–3)_._ Our study data unexpectedly demonstrate that antibody decline in MMF/MPA treated, seroconverted patients, is reduced leading to equivalent seropositivity rates and titers after 6 months of follow-up compared to KTR without MMF/MPA. Our data suggest that MMF/MPA is responsible for a delayed humoral IgG immune response with a different time course specifically of IgG antibody development and decline compared to transplant recipients with immunosuppressive therapy without MMF/MPA, in which 35% were treated with mTOR-I. Almost all KTR with an increasing or *de novo* IgG or RBD-IgG antibody reaction between 2 and 6 months were found in the MMF/MPA group, where this occurred in about a quarter of patients. Interestingly, these MMF/MPA effects were not seen regarding a vaccination-related IgA- or T-cellular response. Whether these results represent an MMF/MPA-mediated problem of a delayed IgM/IgG but not IgA switch remains elusive. A similar delayed immune response in KTR was shown by others after COVID-19 disease (13). Here, an early anti-SARS-CoV-2 IgA and IgM response occurred in KTR, whereas the IgG response appeared delayed compared with immunocompetent individuals. While MMF/MPA similar to other anti-metabolites, such as azathioprine or mTOR-I, exerts a wide array of inhibitory effects on B-, T-, dendritic cells, macrophages, and endothelial cells (14), reduced IgG levels (15) and distinct effects on differential immunoglobulin classes (16), severe differences between MMF/MPA and mTOR-I have been demonstrated in KTR being exposed to either immunocyanin, pneumococcal polysaccharide (PPS), or tetanus toxoid (TT) (17). Hereby, only MMF/MPA severely reduced B-cell numbers and completely disturbed primary and secondary humoral responses, while treatment with the mTOR-I everolimus allowed primary immune responses and boosting of T-cell-dependent and -independent secondary humoral responses to the above vaccines. Nevertheless, vaccination-motivated stop or reduction of MMF/MPA dose and exposure or replacement by mTOR-I need to be balanced with rejection risk. While some transplant centers already consider a temporary stop of MMF/MPA treatment to achieve seroconversion in non-seroconverting KTR, our data demonstrate an MMF/MPA-mediated shift in the antibody time course associated with a decreased risk of decline suggests that this approach is not necessary for seroconverting KTR. In this context, it is interesting that the seroconverted KTR_IgA_ group with IgA but not IgG seroconversion was dominated by MMF/MPA treatment. Within this patient group, mRNA booster (third) vaccinations still led to marked IgG and RBD-IgG titer increases in almost all patients indicating the value of IgA antibody measurements. Nevertheless, despite no clinical consequence of this delayed immune response being visible in our study population, this situation may change dependent on regional pandemic conditions, where timely and strong protection may be required.

Considering the frequency and consequences of insufficient protection in the vulnerable population of transplant recipients, immune monitoring should be an integral part of patient care and used for the timing of additional booster vaccinations. Hereby, MMF/MPA seems to be the most critical drug changing not only the chance of seroconversion but also the peak level and time course of specific IgG and RBD-IgG antibody formation and decline after successful SARS-CoV-2 vaccination.

## Data Availability Statement

After publication of the primary objective, the data might be provided to interested scientists on request (e.g. for meta-analyses, health related registers or other scientific questions) in an anonymized way within five years, if the members of the DIA-Vacc group agree.

## Ethics Statement

According to the professional code of conduct for doctors ($15) the clinical trial was submitted to the ethical institutional review boards at Technische Universität Dresden (TU Dresden) responsible for the coordinating investigator (BO-EK-45012021), as well as at the University of Leipzig (046/21-lk) and Saxon Medical Association (Sächsische Landesärztekammer - EK-BR-10/21-1) responsible for further participating trial sites.

## Author Contributions

Contributors JSt and CH contributed to the study design, data collection, data interpretation, and drafting of the manuscript. TS, JSc, GG, AP, AS, FG, FK, HK, PA, JSr, KF, and TT were involved in data acquisition and collection or study organization. AK and RM were involved in the statistical analysis or data management of the study. TS, JSc, GG, and AP were involved in patient recruitment and data collection. All authors contributed to the article and approved the submitted version.

## Funding

This study was funded by the Else Kröner Fresenius Stiftung, Bad Homburg v. d. H., grant number Fördervertrag EKFS 2021_EKSE.27 and the “Sächsisches Staatsministerium für Wissenschaft, Kultur und Tourismus” *via* Sächsische AufbauBank, grant number (SAB-Antragsnr). (100592538).

## Conflict of Interest

The authors declare that the research was conducted in the absence of any commercial or financial relationships that could be construed as a potential conflict of interest.

## Publisher's Note

All claims expressed in this article are solely those of the authors and do not necessarily represent those of their affiliated organizations, or those of the publisher, the editors and the reviewers. Any product that may be evaluated in this article, or claim that may be made by its manufacturer, is not guaranteed or endorsed by the publisher.

## References

[1] Stumpf J Siepmann T Lindner T Karger C Schwobel J Anders L. Humoral and cellular immunity to SARS-CoV-2 vaccination in renal transplant versus dialysis patients: a prospective, multicenter observational study using mRNA-1273 or BNT162b2 mRNA vaccine. Lancet Reg Health Eur. (2021) 9:100178. 10.1016/j.lanepe.2021.100178 34318288 PMC8299287

[2] Grupper A Rabinowich L Schwartz D Schwartz IF Ben-Yehoyada M Shashar M. Reduced humoral response to mRNA SARS-CoV-2 BNT162b2 vaccine in kidney transplant recipients without prior exposure to the virus. Am J Transplant. (2021) 21:2719–26. 10.1111/ajt.16615 33866672 PMC8250589

[3] Boyarsky BJ Werbel WA Avery RK Tobian AAR Massie AB Segev DL. Antibody response to 2-dose SARS-CoV-2 mRNA vaccine series in solid organ transplant recipients. JAMA. (2021) 325:2204–6. 10.1001/jama.2021.7489 33950155 PMC8100911

[4] Stumpf J Schwobel J Lindner T Anders L Siepmann T Karger C. Risk of strong antibody decline in dialysis and transplant patients after SARS-CoV-2mRNA vaccination: six months data from the observational dia-vacc study. Lancet Reg Health Eur. (2022) 17:100371. 10.1016/j.lanepe.2022.100371 35434688 PMC8995854

[5] Padoan A Sciacovelli L Basso D Negrini D Zuin S Cosma C. IgA-Ab response to spike glycoprotein of SARS-CoV-2 in patients with COVID-19: a longitudinal study. Clin Chim Acta. (2020) 507:164–6. 10.1016/j.cca.2020.04.026 32343948 PMC7194886

[6] Rubio-Acero R Castelletti N Fingerle V Olbrich L Bakuli A Wölfel R. In search for the SARS-CoV-2 protection correlate: a head-to-head comparison of two quantitative S1 assays in a group of pre-characterized oligo-/asymptomatic patients. Infect Dis Ther. (2021) 10:1505–18. 10.1007/s40121-021-00475-x 34137000 PMC8208377

[7] Müller L Andrée M Moskorz W Drexler I Walotka L Grothmann R. Age-dependent immune response to the Biontech/Pfizer BNT162b2 COVID-19 vaccination. Clin Infect Dis. 73:2065–72. 10.1101/2021.03.03.21251066 PMC8135422 33906236

[8] Meyer B Torriani G Yerly S Mazza L Calame A Arm-Vernez I. Validation of a commercially available SARS-CoV-2 serological immunoassay. Clin Microbiol Infect. (2020) 26:1386–94. 10.1016/j.cmi.2020.06.024 32603801 PMC7320699

[9] EUROIMMUN Medizinische Labordiagnostika AG aPc. SARS-CoV-2 NeutraLISA. (2021). Available online at: https://www.coronavirus-diagnostics.com/documents/Indications/Infections/Coronavirus/EI_2606_D_UK_F.pdf (accessed March 31, 2021).

[10] Aiello A Najafi Fard S Petruccioli E Petrone L Vanini V Farroni C. Spike is the most recognized antigen in the whole-blood platform in both acute and convalescent COVID-19 patients. Int J Infect Dis. (2021) 106:338–47. 10.1016/j.ijid.2021.04.034 33864921 PMC8045417

[11] R Core Team. R: A Language and Environment for Statistical Computing. 4.0.1 ed. Vienna: R Foundation for Statistical Computing (2020).

[12] Friedman J Hastie T Tibshirani R. Regularization paths for generalized linear models *via* coordinate descent. J Stat Softw. (2010) 33:1–22. 10.18637/jss.v033.i01 20808728 PMC2929880

[13] Cravedi P Ahearn P Wang L Yalamarti T Hartzell S Azzi Y. Delayed kinetics of IgG, but Not IgA, antispike antibodies in transplant recipients following SARS-CoV-2 infection. J Am Soc Nephrol. (2021) 32:3221–30. 10.1681/ASN.2021040573 34599041 PMC8638399

[14] Ritter ML Pirofski L. Mycophenolate mofetil: effects on cellular immune subsets, infectious complications, and antimicrobial activity. Transpl Infect Dis. (2009) 11:290–7. 10.1111/j.1399-3062.2009.00407.x 19497072 PMC2852585

[15] Jonsson CA Carlsten H. Mycophenolic acid inhibits inosine 5'-monophosphate dehydrogenase and suppresses immunoglobulin and cytokine production of B cells. Int Immunopharmacol. (2003) 3:31–7. 10.1016/S1567-5769(02)00210-2 12538032

[16] Zmonarski SC Boratynska M Madziarska K Klinger M Kusztel M Patrzalek D. Mycophenolate mofetil severely depresses antibody response to CMV infection in early posttransplant period. Transplant Proc. (2003) 35:2205–6. 10.1016/S0041-1345(03)00764-4 14529889

[17] Struijk GH Minnee RC Koch SD Zwinderman AH van Donselaar-van der Pant KA Idu MM. Maintenance immunosuppressive therapy with everolimus preserves humoral immune responses. Kidney Int. (2010) 78:934–40. 10.1038/ki.2010.269 20703211

