# Supplementary material for: MMF/MPA Is the Main Mediator of a Delayed Humoral Response With Reduced Antibody Decline in Kidney Transplant Recipients After SARS-CoV-2 mRNA Vaccination
Source: Front Med (Lausanne). 2022 Jul 7;9:928542. doi: 10.3389/fmed.2022.928542 (PMC9300891; doi:10.3389/fmed.2022.928542)
Supplement: Supplementary file 1 [file Data_Sheet_1.docx]

MMF/MPA is the main mediator of a delayed humoral response with reduced antibody decline in kidney transplant recipients after SARS-CoV-2mRNA vaccination

Supplementary Material

# Supplementary methods

## Clinical data management and data protection

For creation of the study database the EDC tool REDCap (Research Electronic Data Capture)(1, 2) developed and distributed by the Vanderbilt University, has been used. The database has been validated according to the Standard Operating Procedures (SOPs) of the Coordination Centre for Clinical Trials Dresden prior to data capture. The entered data into the eCRF by the investigator or an authorized member of the study team were systematically checked for completeness, consistency and plausibility by routines implemented in the REDCap database such that discrepancies can be dealt with at data entry. Errors and warnings could be resolved at any time during entry process. During the whole course of the study, a backup of all data was made on a daily base. Unauthorized access to patient data was prevented by the access concept of the study database, which is based on a strict hierarchy and role model. Any change of data (e.g. when data is changed in the database during query management) is recorded automatically via audit trail within the database. Any changes to the database are possible only by joint written agreement between coordinating investigator, biometrician and data manager. The Coordination Centre for Clinical Trials Dresden is responsible for implementation of procedures for data collection, storage, protection, retention and destruction. Investigators in the recruiting trial sites initially collected all data. Together with information on the trial, eligible patients and participants were informed about data capture, transmission and analysis processes. Once a participant was eligible and has given his/her informed consent to trial participation and data collection, the investigator has assigned the person a unique patient/participant identification code. This identification code lists are part of the investigator site file and remain at the recruiting site. These lists are the only documents that allow for re-identification of the patients. Participant data were recorded in pseudonymized form (i.e. without reference to the patient’s name) using the identification code. Data capture and processing was in accordance with the applicable law 6 on personal data protection and with the “General Data Protection Regulation” (EC) 2016/679 of the European parliament and of the council.

# Supplementary tables

Table S1: Baseline characteristics of SARS-CoV-2 unexposed, but at T2 seroconverted persons / patients of the KTR_112_ vaccination cohort without/with MMF/MPA treatment

| ***Variable*** | ***Category*** | ***KTR without MMF/MPA*** | ***KTR with MMF/MPA*** |
| --- | --- | --- | --- |
| ***Number*** | evaluable | 51 | 61 |
| ***Age (years)*** | mean ± SD | 59.1 ± 12.6 | 56 ± 13 |
| ***Male Sex*** | n / % | 29 / 56.9 | 40 / 65.6 |
| ***BMI (kg/m^2^)*** | mean ± SD | 26.4 ± 5 | 26.5 ± 4.5 |
| ***Cause of end stage renal disease*** | n / % | 29 / 56.9 | 43 / 70.5 |
| Diabetes-Hypertension-Vascular disease | n / % | 6 / 11.8 | 13 / 21.3 |
| Glomerulonephritis-Interstitial nephritis | n / % | 13 / 25.5 | 16 / 26.2 |
| Vasculitis | n / % | 1 / 2 | 2 / 3.3 |
| Polycystic kidney disease | n / % | 9 / 17.6 | 12 / 19.7 |
| Unknown | n / % | 22 / 43.1 | 18 / 29.5 |
| ***Drug treated comorbidities*** | n / % | 42 / 82.4 | 55 / 90.2 |
| Diabetes mellitus | n / % | 9 / 17.6 | 15 / 24.6 |
| Cardiovascular disease | n / % | 40 / 78.4 | 52 / 85.2 |
| Lung disease | n / % | 2 / 3.9 | 4 / 6.6 |
| Liver cirrhosis | n / % | 0 / 0 | 1 / 1.6 |
| Cancer | n / % | 3 / 5.9 | 2 / 3.3 |
| None | n / % | 9 / 17.6 | 6 / 9.8 |
| ***Time on dialysis (years)*** | *mean ± SD* | 6.8 ± 8.2 | 5.6 ± 3.8 |
| ***On transplant waiting list*** | *n / %* | 0 / 0 | 0 / 0 |
| ***Time on transplantation (years)*** | *mean ± SD* | 12.1 ± 8.5 | 11.5 ± 7.2 |
| ***Previous transplantation*** | n / % | 12 / 23.5 | 7 / 11.5 |
| ***Hepatitis B vaccination failure*** | n / % | 4 / 7.8 | 7 / 11.5 |
| ***Flu vaccination winter 2020/21*** | n / % | 27 / 52.9 | 33 / 54.1 |
| ***On immunosuppressive therapy*** | n / % | 50 / 98 | 61 / 100 |
| Corticosteroids | n / % | 24 / 47.1 | 22 / 36.1 |
| Calcineurin-Inhibitor | n / % | 40 / 78.4 | 54 / 88.5 |
| MMF/MPA | n / % | 0 / 0 | 61 / 100 |
| mTOR-Inhibitor | n / % | 18 / 35.3 | 5 / 8.2 |
| Belatacept | n / % | 2 / 3.9 | 1 / 1.6 |
| T-cell depleting ab | n / % | 0 / 0 | 0 / 0 |
| B-cell depleting ab | n / % | 0 / 0 | 0 / 0 |
| Other | n / % | 4 / 7.8 | 0 / 0 |
| ***Type of vaccine*** |  |  |  |
| BNT162b2 mRNA | n / % | 16 / 31.4 | 14 / 23 |
| mRNA-1273 | n / % | 35 / 68.6 | 47 / 77 |

*For this evaluation all patients with asymptomatic* or documented symptomatic** COVID-19 disease before and during vaccination or with a third vaccination* *up to T3 (six months) were excluded. Hepatitis B vaccination failure definition - patients with unsuccessful vaccination after at least four attempts; KTR = Kidney Transplant Recipient*; *MMF/MPA = mycophenolate mofetil or mycophenolic acid;* *mRNA-1273 represents Spikevax also called Moderna COVID-19 vaccine; BNT162b2-mRNA which stands for Comirnaty also known as Pfizer-BioNTech COVID-19 vaccine;*

**Asymptomatic COVID-19 disease definition - neither knowledge nor symptoms of COVID-19 disease, but IgG-antibody reaction to nucleocapsid (T0, T1, T2, or T3) or to the Spike protein subunit S1 (only T0) of the SARS-CoV-2 virus is positive.*

***Symptomatic COVID-19 disease definition - SARS-CoV-2 PCR positive patients with clinical symptoms;*

Table S2: Interval categorization of IgG and RBD ranges

| IgG Level | Interval [BAU/ml] | Participants at T2 (n) | Participants at T3 (n) |
| --- | --- | --- | --- |
| 0 | IgG < 35·2 | 6 | 18 |
| 1 | 35·2 ≤ IgG < 100 | 19 | 30 |
| 2 | 100 ≤ IgG < 200 | 18 | 24 |
| 3 | 200 ≤ IgG < 300 | 9 | 12 |
| 4 | IgG ≥ 300 | 60 | 28 |
| RBD Level | **Interval [% IH]** | **Participants at T2 (n)** | **Participants at T3 (n)** |
| 0 | RBD < 35 | 20 | 41 |
| 1 | 35 ≤ RBD < 50 | 16 | 17 |
| 2 | 50 ≤ RBD < 65 | 10 | 11 |
| 3 | 65 ≤ RBD < 80 | 14 | 11 |
| 4 | RBD ≥ 80 | 46 | 26 |

*BAU/ml means binding antibody units per mililiter; % IH means % inhibition;*

Table S3: Antibody response (titer) in solely IgA-seroconverted, MMF/MPA treated KTR_IgA_ cohorts without/with third mRNA vaccination

| *Variable* | *Time* | *KTR_IgA_ (unboostered)* | *KTR_IgA_ (boostered)* |
| --- | --- | --- | --- |
| Patient number |  | 5 | 19 |
| IgA-Ab Spike S1 | T2 | 1.8 (1.4 - 3.2) | 1.8 (1.1 - 3) |
| IgA-Ab Spike S1 | T3 | 0.5 (0.5 - 1.2) | 2 (1.6 - 6) |
| IgG-Ab Spike S1 | T2 | 6 (6 - 6.3) | 13.8 (4.1 - 26.5) |
| IgG-Ab Spike S1 | T3 | 17.1 (5.4 - 62.5) | 297.3 (75.4 - 384) |
| RBD-IgG | T2 | 20.9 (20.9 - 20.9) | 8.1 (4.7 - 12.9) |
| RBD-IgG | T3 | 4 (1.9 - 17.8) | 90.8 (30.6 - 99) |

*KTR = Kidney Transplant Recipient;*

# Supplementary figures

Figure S1: Study schedule

**
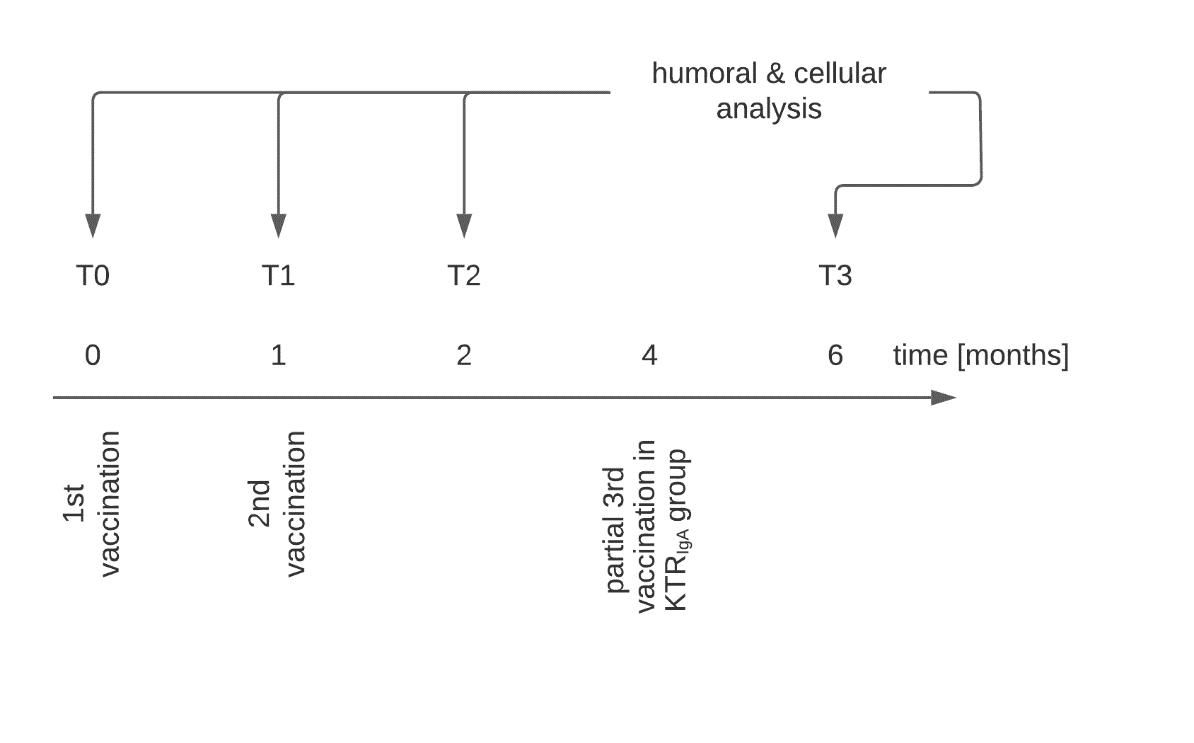
***T0, T1, T2 and T3 = corresponding time points at month 0, 1, 2, and 6. KTR_IgA_ subgroup consists of anti S1 IgA antibody only seroconverted kidney transplant recipients after 1st and 2nd vaccination.*

Figure S2A: IgG distribution at T2 in KTR_112_ without (green) and with (orange) MMF/MPA


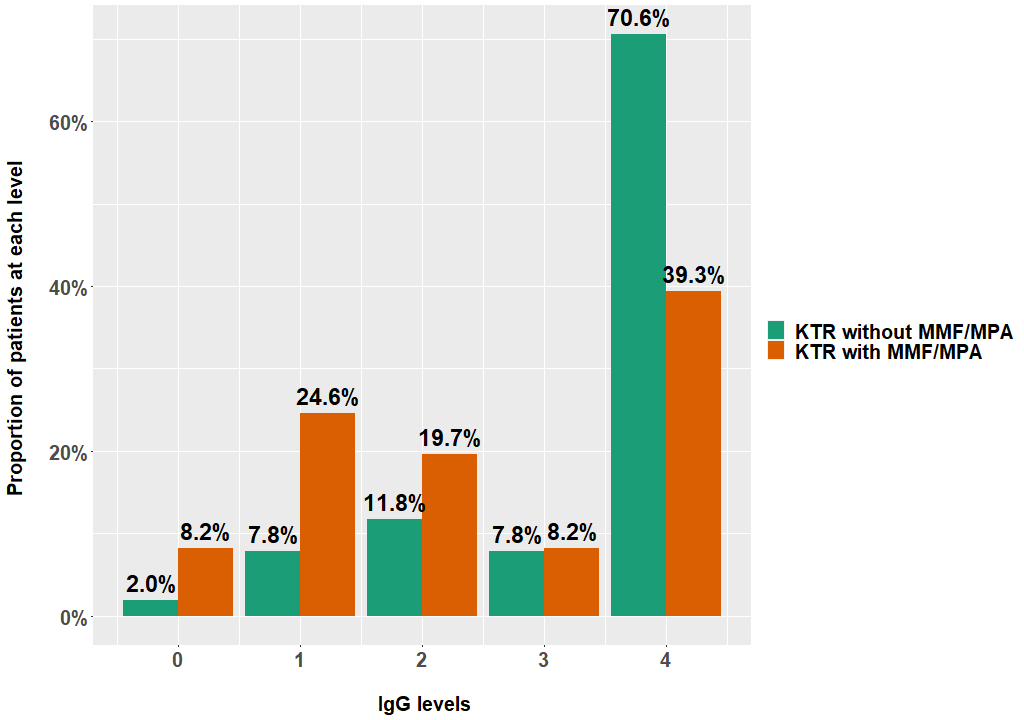
*The distributions of anti-SpikeS1 IgG levels according to interval categorizations at T2 in kidney transplant recipients (KTR). Level “0” is assigned to values below the corresponding positivity threshold (IgG < 35.2 binding antibody units per milliliter [BAU/ml]). The remaining test values were divided into four intervals of approximately equal length (level “1” < 100 and ≥ 35.2; level “2” < 200 and ≥ 100; level “3” < 300 and ≥ 200; level “4” ≥ 300 BAU/ml). Kidney transplant recipients (KTR) taking the immunosuppressant mycophenolate mofetil or mycophenolic acid (MMF/MPA) are shown in orange whereas those without MMF/MPA are depicted in green.*

Figure S2B: IgG distribution at T3 in KTR without (green) and with (orange) MMF/MPA


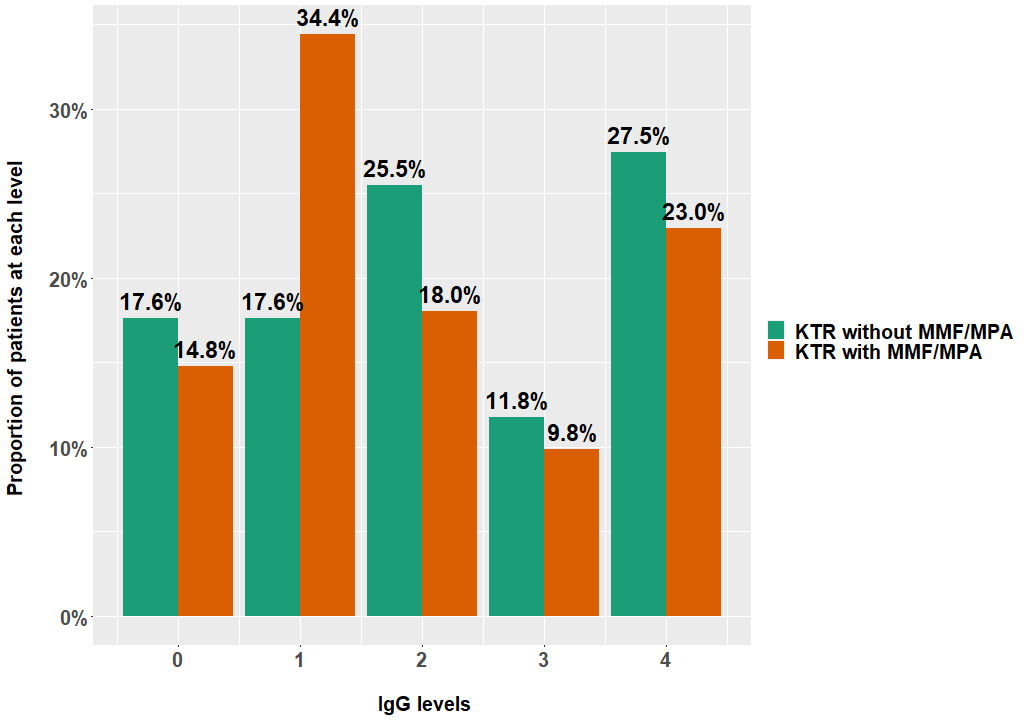
*The distributions of anti-SpikeS1 IgG levels according to interval categorizations at T3 in kidney transplant recipients (KTR). Level “0” is assigned to values below the corresponding positivity threshold (IgG < 35.2 binding antibody units per milliliter [BAU/ml]). The remaining test values were divided into four intervals of approximately equal length (level “1” < 100 and ≥ 35.2; level “2” < 200 and ≥ 100; level “3” < 300 and ≥ 200; level “4” ≥ 300 BAU/ml). Kidney transplant recipients (KTR) taking the immunosuppressant mycophenolate mofetil or mycophenolic acid (MMF/MPA) are shown in orange whereas those without MMF/MPA are depicted in green.*

Figure S2C: Distribution of IgG level change between T2 and T3 in KTR_112_ without (green) and with (orange) MMF/MPA

**
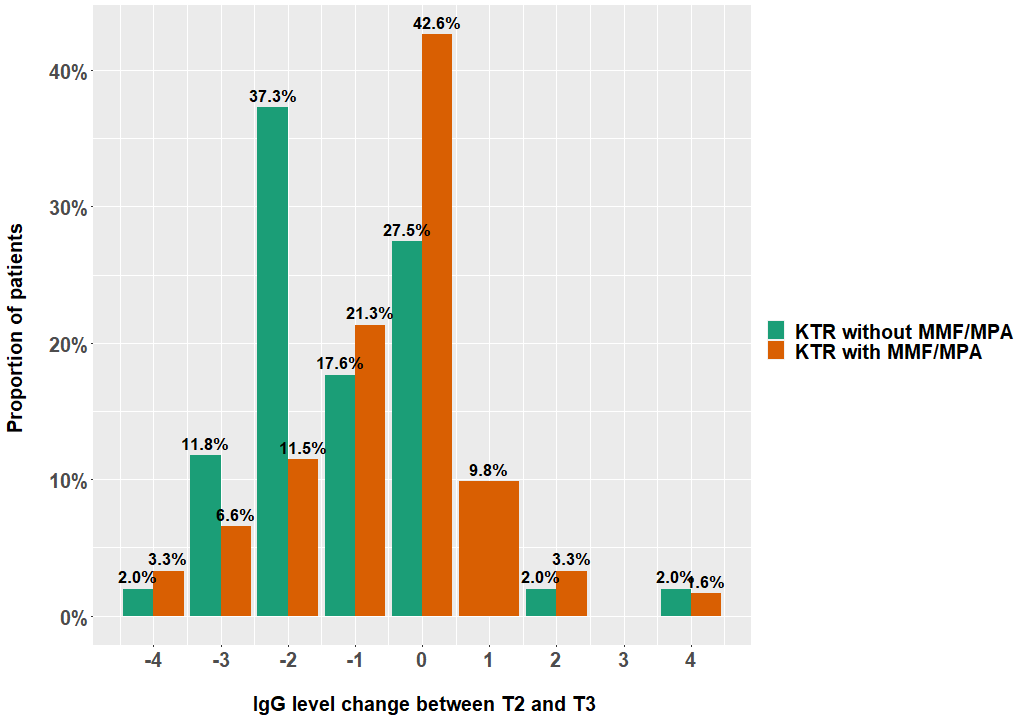
***The distributions of anti-SpikeS1 IgG level change between T2 and T3 according to interval categorizations at these very times in kidney transplant recipients (KTR). Level changes (proportions of patients) between T2 and T3 in KTR taking the immunosuppressant mycophenolate mofetil or mycophenolic acid (MMF/MPA), shown in orange, versus in green without this immunosuppressant.*

Figure S2D: RBD-IgG distribution at T2 in KTR_112_ without (green) and with (orange) MMF/MPA

***
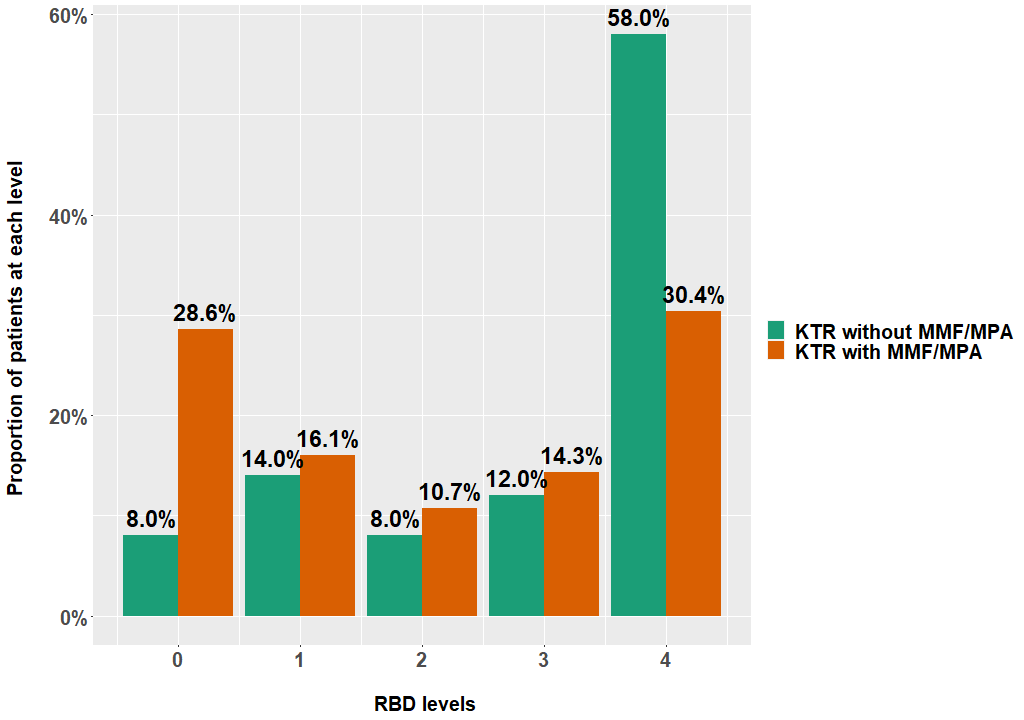
****The distributions of anti-RBD IgG levels according to interval categorizations at T2 in kidney transplant recipients (KTR). Level “0” is assigned to values below the corresponding positivity threshold (RBD < 35 % inhibition [IH]). The remaining test values were divided into four intervals of approximately equal length (level “1” < 50 and ≥ 35; level “2” < 65 and ≥ 50; level “3” < 80 and ≥ 65; level “4” ≥ 80 % IH). Kidney transplant recipients (KTR) taking the immunosuppressant mycophenolate mofetil or mycophenolic acid (MMF/MPA) are shown in orange whereas those without MMF/MPA are depicted in green.*

Figure S2E: RBD-IgG distribution at T3 in KTR_112_ without (green) and with (orange) MMF/MPA

***
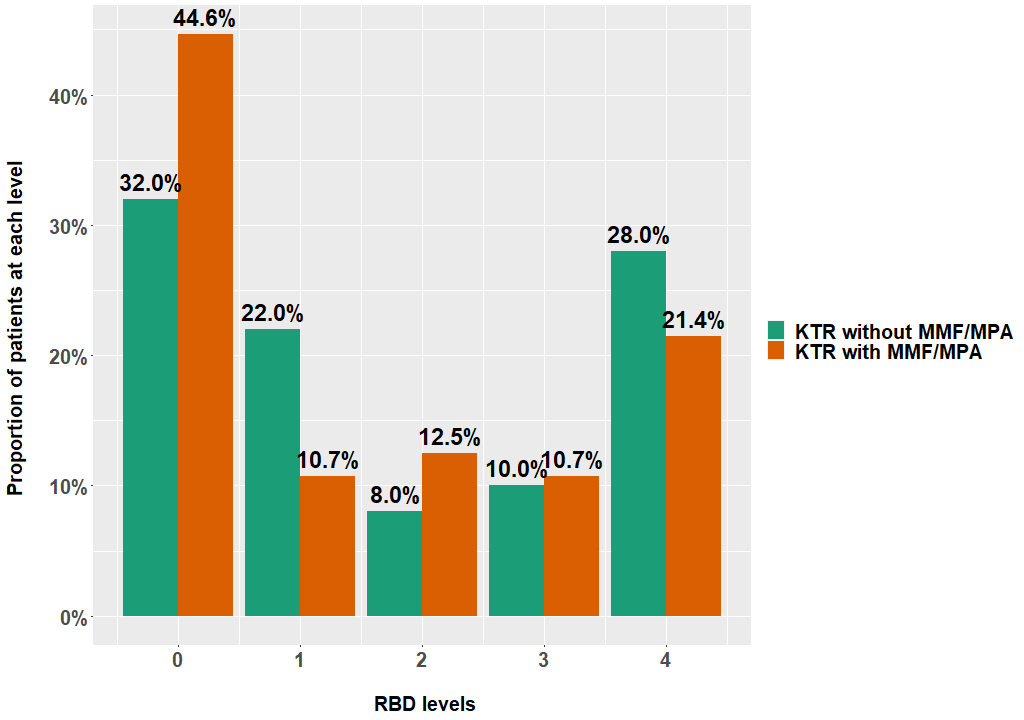
****The distributions of anti-RBD IgG levels according to interval categorizations at T3 in kidney transplant recipients (KTR). Level “0” is assigned to values below the corresponding positivity threshold (RBD < 35 % inhibition [IH]). The remaining test values were divided into four intervals of approximately equal length (level “1” < 50 and ≥ 35; level “2” < 65 and ≥ 50; level “3” < 80 and ≥ 65; level “4” ≥ 80 % IH). Kidney transplant recipients (KTR) taking the immunosuppressant mycophenolate mofetil or mycophenolic acid (MMF/MPA) are shown in orange whereas those without MMF/MPA are depicted in green.*

Figure S2F: Distribution of RBD-IgG level change between T2 and T3 in KTR_112_ without (green) and with (orange) MMF/MPA

***
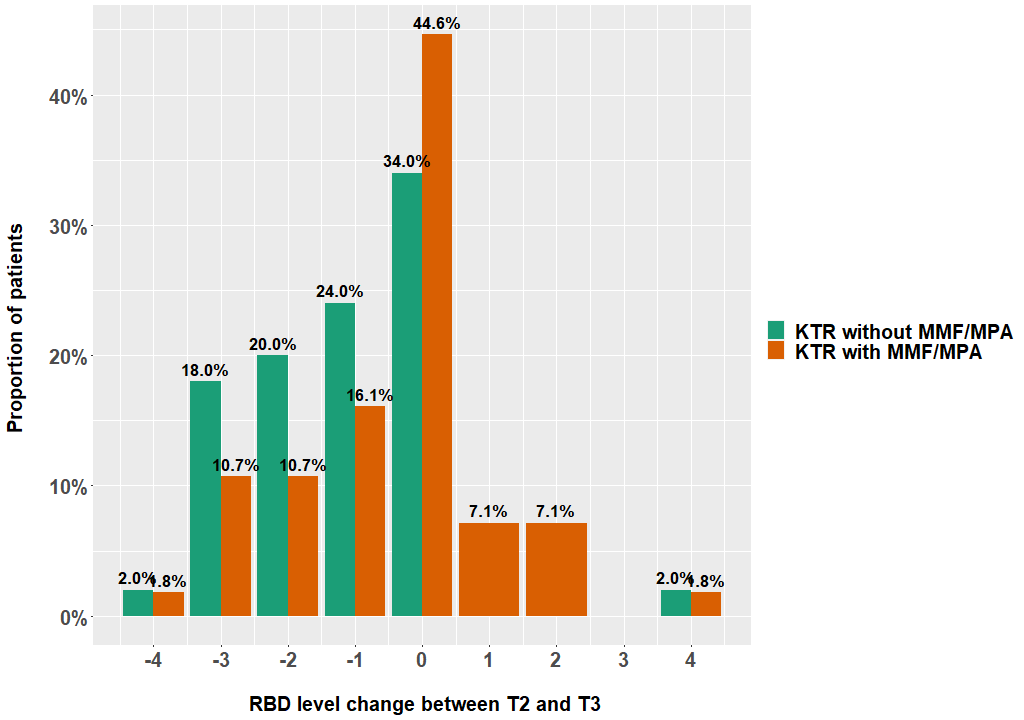
****The distributions of anti-RBD IgG level change between T2 and T3 according to interval categorizations at these very times in kidney transplant recipients (KTR). Level changes (proportions of patients) between T2 and T3 in KTR taking the immunosuppressant mycophenolate mofetil or mycophenolic acid (MMF/MPA), shown in orange, versus in green without this immunosuppressant.*

Figure S3: A penalized logistic regression model estimated using the elastic net approach to analyze the association between immunosuppression type and strong declining IgG response

**
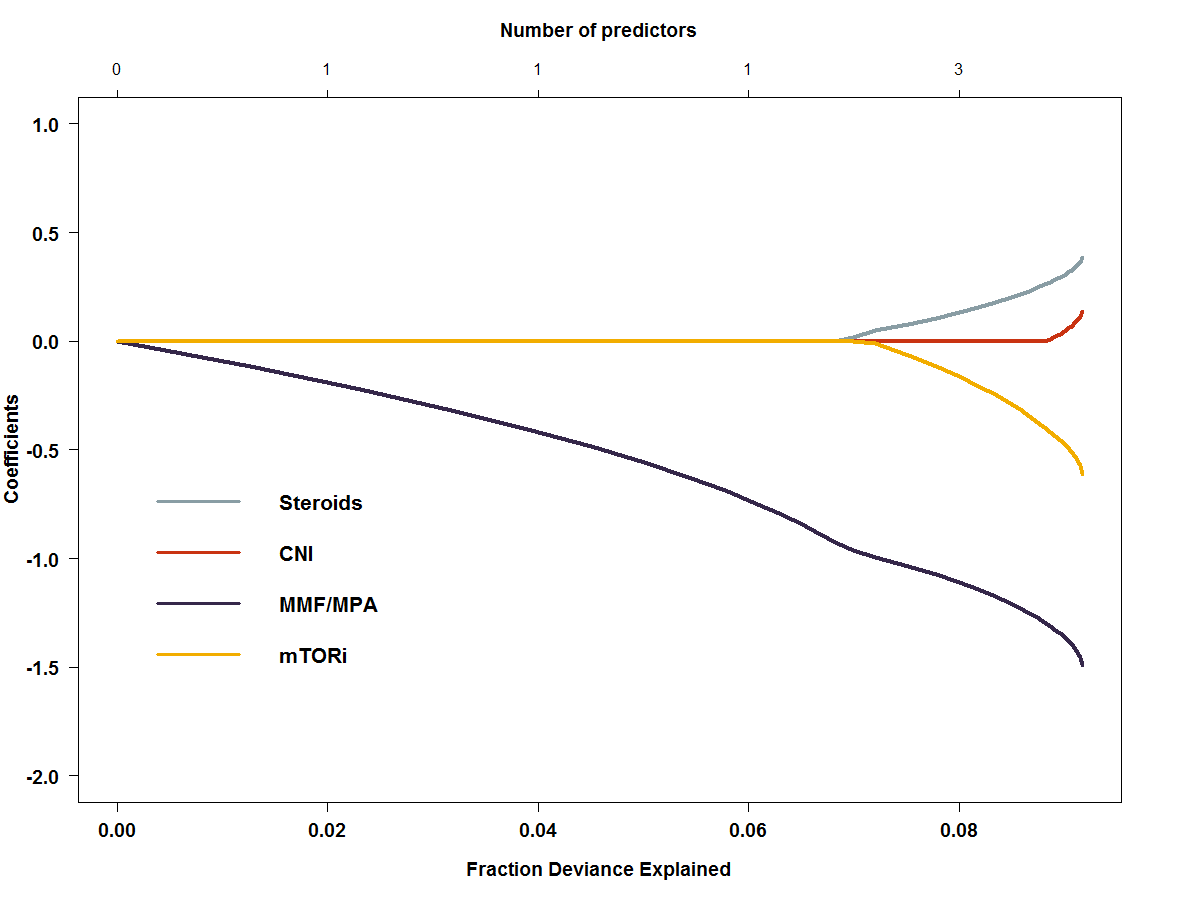
***A stepwise model selection procedure is shown in which predictors (4 immunosuppressive drug types) are added to a regression model one at a time, to maximize the goodness-of-fit, assessed from the deviance, given the current number of predictors. The slope of each path in the figure changes as a new IS drug enters the model. According to this plot, MMF/MPA has the strongest explanatory ability as a single predictor.*

Figure S4: Time course of anti-SARS-CoV-2 IgG antibodies in solely IgA-seroconverted, MMF/MPA treated KTR_IgA_ cohorts without (green) or with (orange) third mRNA vaccination


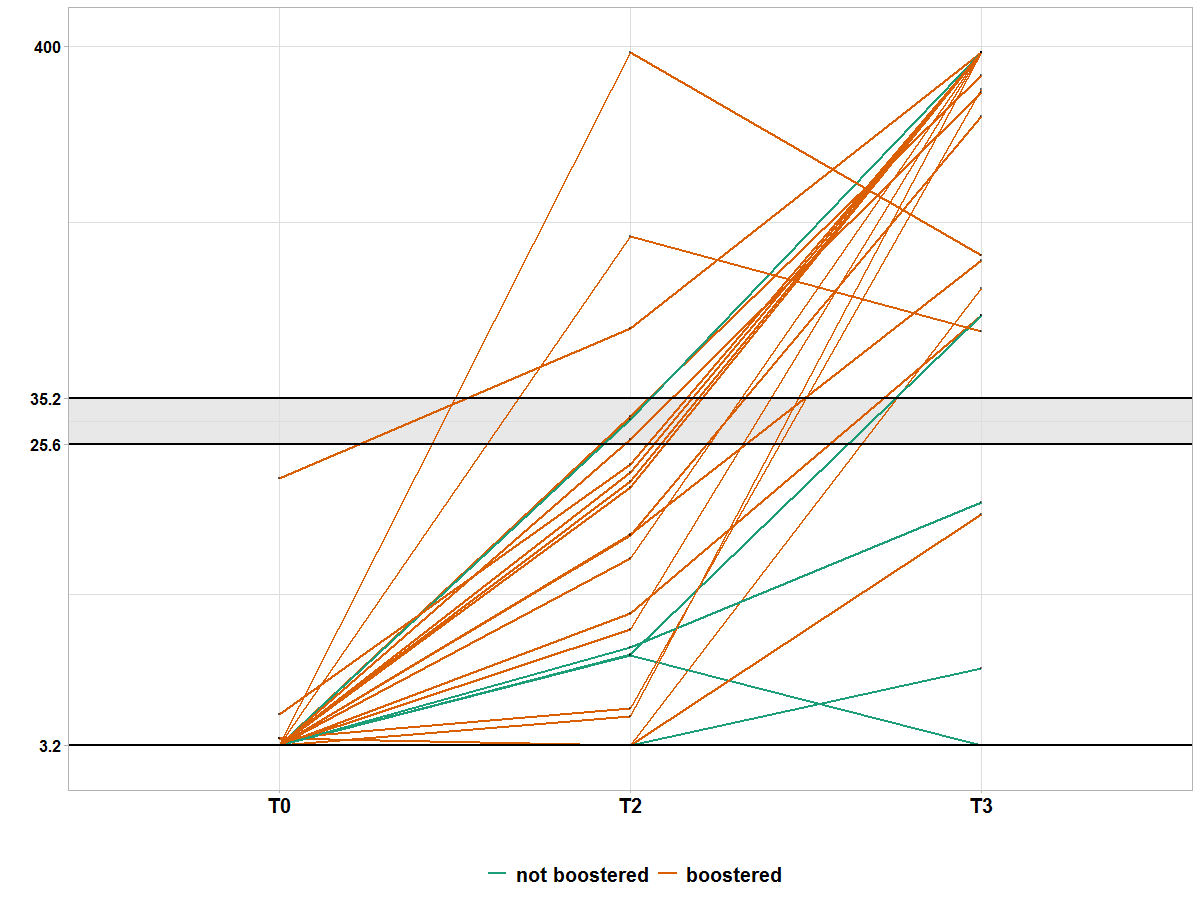
*Each thin line corresponds the anti-Spike S1 protein IgG antibody values (QuantiVac, Euroimmun) of a MMF/MPA treated study participant from T0 (vaccination start) via T2 (eight weeks after vaccination start) to T3 (six months after vaccination start). KTR being not vaccinated a third time are represented in green and KTR being vaccinated a third time at 4.2±1* *months are shown in orange. Only patients with de novo seroconversion of IgA but not IgG antibodies against the SARS-CoV-2 S1 protein at T2 after 2x mRNA vaccination and without SARS-CoV-2 nucleocapsid (NCP) antibodies were considered. The area shaded grey designates IgG borderline range below positivity level. The vertical axis is depicted on* ${log}_{10}$ *scale with corresponding unit BAU/ml.*

# Supplementary bibliography

1. Harris PA, Taylor R, Thielke R, Payne J, Gonzalez N, Conde JG. Research electronic data capture (REDCap)--a metadata-driven methodology and workflow process for providing translational research informatics support. J Biomed Inform. 2009;42(2):377-81.

2. Harris PA, Taylor R, Minor BL, Elliott V, Fernandez M, O'Neal L, et al. The REDCap consortium: Building an international community of software platform partners. J Biomed Inform. 2019;95:103208.

# Data Availability Statement

## Declaration regarding data sharing

After publication of the primary objective, the data might be provided to interested scientists on request (e.g. for meta-analyses, health related registers or other scientific questions) in an anonymized way within five years, if the members of the DIA-Vacc group agree.
